# Perceived quality of life, fatigue and the metabolic cost of walking in generalized hypermobility spectrum disorder and hypermobile Ehlers-Danlos syndrome

**DOI:** 10.3389/fresc.2025.1706912

**Published:** 2025-11-28

**Authors:** Aera J. M. Ladell, Donald W. Golden, Jason P. Oliemans, Kalindra D. Walls, Ranita H. K. Manocha, Jared R. Fletcher

**Affiliations:** 1 Department of Biology, Mount Royal University, Calgary, AB, Canada; 2 Faculty of Kinesiology, University of Calgary, Calgary, AB, Canada; 3 Department of Health and Physical Education, Mount Royal University, Calgary, AB, Canada; 4 Cumming School of Medicine, University of Calgary, Calgary, AB, Canada

**Keywords:** SF-36, foot function, fatigue, hypermobility, Ehlers-Danlos syndrome

## Abstract

**Introduction:**

Individuals with hypermobile Ehlers-Danlos Syndrome (hEDS) and Hypermobility Spectrum Disorders (HSD) often experience chronic pain, leg fatigue and/or pain, which may contribute to a higher metabolic cost of walking. How these subjective measures may manifest in an elevated metabolic cost of transport and perceived pain at varying walking speeds remains to be evaluated.

**Methods:**

We recruited 11 HSD/hEDS participants (33 ± 14 yrs, 170 ± 6. cm, weight 71 ± 11 kg) and 11 age- and sex-matched controls. Self-reported subjective outcome measures of health-related quality of life, fatigue severity, and foot function were evaluated prior to treadmill walking using the 36-Item Short Form Health Survey (SF-36) and the Fatigue Severity Scale (FSS). Participants also rated their self-perceived leg pain/fatigue following 3 x 6 min of treadmill walking at 80%, 100% and 120% of preferred walking speed (PWS). Cost of transport (CoT) was calculated from indirect calorimetry.

**Results:**

CoT was significantly higher in HSD/hEDS compared to CON at all speeds (*p* = 0.034). CoT was significantly higher at 80% PWS compared to both 100% and 120% PWS in HSD/hEDS. HSD/hEDS reported significantly poorer health outcomes across all SF-36 domains (*p* < 0.05), significantly poorer foot function (*p* < 0.001) and significantly higher fatigue severity (*p* < 0.001) prior to walking. Despite similar PWS (1.1 ± 0.3 m⸱s^-1^), Leg pain/fatigue immediately following walking was significantly higher in HSD/hEDS compared to CON (group main effect *p* < 0.001) and increased with speed in HSD/hEDS (p = 0.011). The physical function domain was significantly related post-walking leg pain/fatigue, and perceived energy prior to walking (all *r* > 0.69, *p* < 0.001).

**Discussion:**

These results have important implications for exercise prescription in individuals with HSD/hEDS whose CoT is higher during walking, which may exacerbate already elevated symptoms of pain and fatigue.

## Introduction

Hypermobile Ehlers-Danlos syndrome (hEDS) is a heritable connective tissue disorder often characterized by generalized joint hypermobility, joint hypermobility, joint dislocations/subluxations) and minor skin manifestations (velvety, soft, feel and mild skin hyperextensibility), as well as dysautonomia, chronic pain and fatigue (1). The clinical criteria for hEDS diagnosis firstly include generalized joint hypermobility, indicated by a Beighton score ≥ 5/9 (2). Hypermobility spectrum disorders (HSD) describe a collection of phenotypes related to joint hypermobility that do not meet the criteria for hEDS or another connective tissue disorder.

Fatigue generally can be considered a subjective experience of feeling exhausted, tired, weak or having lack of energy (3), and is common in HSD/hEDS (4). In this context, fatigue differs greatly from the typical definition in the healthy population compared to pathological fatigue in that it is not necessarily a response to intense physical exertion and is not predictable or necessarily transient. Fatigue in the HSD/hEDS population is also detrimental to emotional, social, and occupational well-being (5). It is therefore important to quantify subjective fatigue prior to exercise in order to better understand if and how this fatigue may manifest following exercise.

The level of physical fitness has been widely documented in a variety of clinical populations to be important in prevention of injury, maintaining physical function and activities of daily living and reducing the perception of fatigue (6, 7). Physical activity and exercise improve, among other factors, the experience of chronic pain, increases physical function, improves sleep, improves cognitive function, and increased overall health (8, 9).

In HSD/hEDS, there are three main barriers to exercise: pain, fatigue and fear of injury, yet exercise is considered a foundational treatment for management of HSD/hEDS (10). If we accept the psychobiological model of fatigue (11), which posits exercise is tolerated to some fixed level of perceived exertion (12, 13), exercise duration at a fixed intensity may be limited in HSD/hEDS because of a higher initial perception of effort. Similarly, perceived fatigue may be higher in HSD/hEDS following a fixed-duration exercise at the same intensity. In HSD/hEDS, exercise intensity is often prescribed based on some rating of perceived exertion (14, 15), similar to how exercise intensity is prescribed in individuals without HSD/hEDS. This prescription, however, ignores the potential differences in ‘resting’ or baseline pain and/or fatigue in individuals with HSD/hEDS. As such, exercise prescription may be misprescribed in HSD/hEDS based on these perceived exertional criteria if resting or baseline fatigue is not known.

There are several valid, and reliable questionnaires to address patient self-perceived quality of life and subjective ratings of fatigue prior to exercise. The 36-item Short Form (SF-36) is a health-related quality of life survey developed for clinical use, public health research, health policy evaluations and general population surveys. The SF-36 is a commonly used diagnostic tool for measuring quality of life and has been previously validated in both healthy and HSD/hEDS populations (16). The SF-36 assesses eight specific health parameters: (1) physical limitations due to health issues, (2) social limitation dues to health-related physical and/or emotional issues, (3) role limitations due to physical health issues, (4) bodily pain, (5) general mental health, 6) role limitations due to emotional issues, (7) energy and fatigue, and 8) general health issues (17, 18). A systematic review and meta-analysis conducted by Umar et al. (19) demonstrated that individuals with any form of Ehlers-Danlos Syndrome (not simply HSD/hEDS) scored significantly lower in all health-related quality of life aspects assessed by the SF-36 compared to the general population, with the most pronounced disparity found within the physical component of well-being. In a previous study (20), we showed that reported pain following walking was higher in HSD/hEDS. How these subjective metrics of physical well-being, fatigue and foot function measured *prior to* walking relate to manifestations of fatigue and/or pain *immediately following* walking remains to be seen. Therefore, the primary objective of this study was to determine if self-reported pain was exacerbated in individuals with HSD/hEDS following walking. A secondary purpose was to quantify the relationship between self-perceived quality of life and subjective fatigue prior to walking and lower leg pain/fatigue following walking.

## Methods

### Participants

Individuals with HSD/hEDS were recruited from a tertiary physiatry clinic focused on the musculoskeletal consequences of HSD/hEDS. Participants were included if they were previously diagnosed with hEDS (*n* = 10) or met the criteria for generalized joint HSD (G-HSD) (*n* = 1) (21). Additional inclusion criteria included participants being able to walk unassisted for at least 30 min and not have a history of smoking or be taking medication that prevented them from being able to complete the walking trial or had a positive response to the Physical Activity Readiness Questionnaire (22). The data presented here represents a secondary analysis of the subjective, quality of life, foot function and fatigue severity data collected during the course of a previously-published study from our lab (20), the purpose of which was to determine how muscle and tendon mechanics contribute to a higher metabolic cost of walking in HSD/hEDS.

Following recruitment of an HSD/hEDS participant, age- and sex-matched individuals without HSD/hEDS were recruited to serve as controls (CON). These otherwise healthy individuals had a Beighton score <4/9 with no prior cardiovascular, neurological, or musculoskeletal disorders. Participants provided informed, written consent to the experimental procedures, which were approved by the Human Research Ethics Board at Mount Royal University (HREB ID#102279). As this is a secondary use of data from the study by Sheehan et al. (22), participant characteristics are the same (Table 1). Participants were asked to complete a baseline FFI, FSS and SF-36 inspired questionnaire prior to their visit to the laboratory. The original SF-36 investigates eight domains of health-related quality of life (17). For this study, we developed a questionnaire inspired from the original SF-36. In this questionnaire, we examined five domains related to pain and fatigue. The other three domains related to mental and social health associated with the original SF-36 were beyond the scope of this study, thus were not considered by the participants. Therefore, the five domains that were included were: (1) physical limitations due to health issues, (2) role limitations due to physical health issues, (3) bodily pain, (4) energy and fatigue, and (5) general health issues. Responses to each SF-36 item were coded from 0 to 100 in 10, 20, or 25-point increments such that a high score indicates a more favorable health state. Responses within each domain were averaged to create an average score for each domain for each participant. Three additional questions were added to the SF-36 that related specifically to the HSD/hEDS population, although these questions could also be used in other clinical populations that experience pain and fatigue. Question 17 (*how much does your heath limit your ability to keep balance*) was categorized into the physical limitations domain. Question 25 (‘*I am fearful I will injure myself*’) and question 28 (*I feel discouraged about my physical health*’) were categorized into the energy and fatigue domain (23). The adapted SF-36 used for this study can be found in Supplementary Materials.

**Table 1:** Participant characteristics.

Characteristic	HSD/hEDS	CON
Age (years)	33.2 ± 14.0	32.6 ± 14.1
Sex	*n* = 10 female; *n* = 1 male	
Height (cm)	169 ± 6	166 ± 10
Weight (kg)	70.6 ± 11.0	67.4 ± 14.6
Beighton Score	6.8 ± 1.1	0.9 ± 0.9*
PWS (m·s^−1^)	1.04 ± 0.31	1.17 ± 0.27

Values are mean ± SD. PWS: preferred walking speed.

* Significantly different between groups (*p* < 0.05). Modified from (20).

A modified Fatigue Severity Scale (FSS) was used to assess participant self-reported impact of fatigue on daily function. The FSS is a 9-item, 7-point Likert scale questionnaire where higher scores indicate greater fatigue severity (24). For easy interpretation of the scale for our participants, we used a modified 10-point Likert scale questionnaire from 1 (‘strongly disagree) to 10 (strongly agree) as anchor statements. In addition, baseline global fatigue was assessed using a 10-point Likert scale from 0 (‘worst fatigue’) to 10 (‘no fatigue’). Similarly, we utilized a modified version of the FFI, specific to HSD/hEDS populations to assess self-perceived foot function and disability related to the foot which can be found in Supplementary Material (25).

Following completion of the SF-36, FFI and FSS, participants walked on a motorized treadmill (Woodway Pro, Woodway USA, Waukeshka, WA) with no gradient for 10 min, as previously described by Sheehan et al. (20). Briefly, the treadmill speed was increased and decreased at least three times to determine the participant's preferred walking speed (PWS) (26). Participants then performed three walking trials in a randomized order at 80, 100% and 120% PWS for six minutes with a minimum of a two minute seated recovery period between walking trials. In the final two minutes of walking at each speed, expired V̇O_2_ and V̇CO_2_ were measured using a metabolic cart (Quark CPET, Cosmed, Rome, Italy). The metabolic cart was calibrated using room air and a gas mixture of known composition (5% CO_2%_ and 16% O_2_) prior to each testing session. The turbine and flow sensor were manually calibrated with a 3 L syringe. Expired gases were collected through the metabolic mask for the entire duration of each trial. A steady state V̇O_2_ (defined as a change of <150 ml/min for any 10s period) was achieved for all participants during the last two minutes of each trial.

The cost of transport (CoT) was calculated from the average steady-state V̇O_2_ and V̇CO_2_ at each speed according to Péronnet and Massicotte (27), and expressed as a relative energy cost per unit distance (J·kg^−1^·m^−1^):

$$\begin{array}{rl} CoT(J\cdot kg^{-1}\cdot m^{-1})\; & =\;16.89\dot{V}O_{2}\;+\;4.84\dot{V}CO_{2}*\;BM^{-1}\;*\;\;s^{-1}\;\\ & \times \;1000 \end{array}$$

where *V̇O_2_* and *V̇CO_2_* are in L*·*s^−1^, *BM* is body mass (in kg), *s* is speed (in m*·*s^−1^) and 1,000 J*·*kJ^−1^. Between trials, participants recovered by sitting or standing quietly for at least five minutes.

Immediately following the completion of each walking trial, a 100 mm visual analog scale (VAS) was used to rate each participant's self-reported leg pain/fatigue. The VAS was a continuous scale depicted as a 100 mm horizontal line, with anchor statements of “*no lower leg pain/fatigue as a result of the walking trial*” (0 mm) and “*worst lower leg pain and/or fatigue imaginable as a result of the walking trial*” (100 mm), respectively. Participants were reminded by a study investigator of these anchor statements, with particular emphasis on lower leg pain/fatigue following the completion of each walking trial.

### Statistical analysis

Values are presented as mean ± standard deviation (SD), unless otherwise indicated. Differences between groups were assessed using independent sample *t*-tests. A two-way (group x speed) repeated measures analysis of variance (ANOVA) was used to evaluate differences in VAS within speeds, and between groups. Normality and equality of variance for all dependent variables were assessed using Shapiro–Wilk test, and Mauchly's test of sphericity test, respectively. When the assumption of sphericity was violated, a Greenhouse-Geisser correction was performed to correct for the Type I error rate. Where a significant main effect of speed or group was found, Holm's *post hoc* tests were used to test for difference between speeds or groups, respectively. The relationship between physical function (SF-36), body pain (SF-36), energy/fatigue (SF-36) and the VAS rating following walking at the preferred speed was determined using Pearson Product-Moment correlation analysis. The relationship between the change in lower leg pain/fatigue at faster (i.e., 120% PWS) and slower (i.e., 80% PWS) walking speeds, expressed as a %change relative to the change in CoT at these speeds, also expressed as a %change, was also calculated using a Pearson Product-moment analysis.

All statistical analyses were conducted in in JASP (v.0.19.0). The *a priori* level of statistical significance was set at *p* < 0.05.

## Results

**Figure 1:**
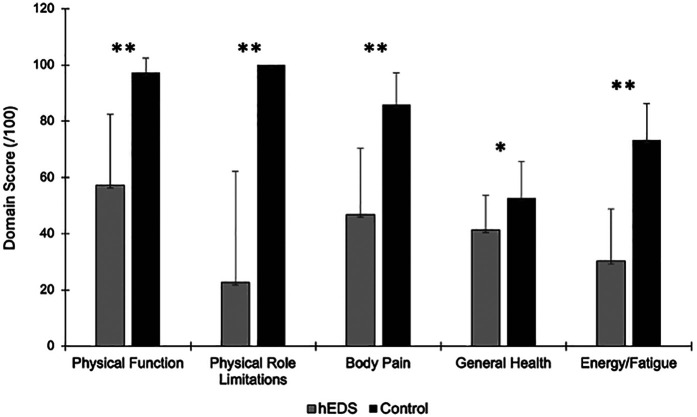
Mean ± SD FSS for HSD/hEDS (grey) and CON (black). * indicates significant group differences (*p* < 0.05), ** indicates significant group differences (*p* < 0.001).

Participant characteristics are shown in Table 1. In all SF-36 domains, HSD/hEDS were significantly worse than CON (*p* < 0.001, Figure 1).

**Figure 2:**
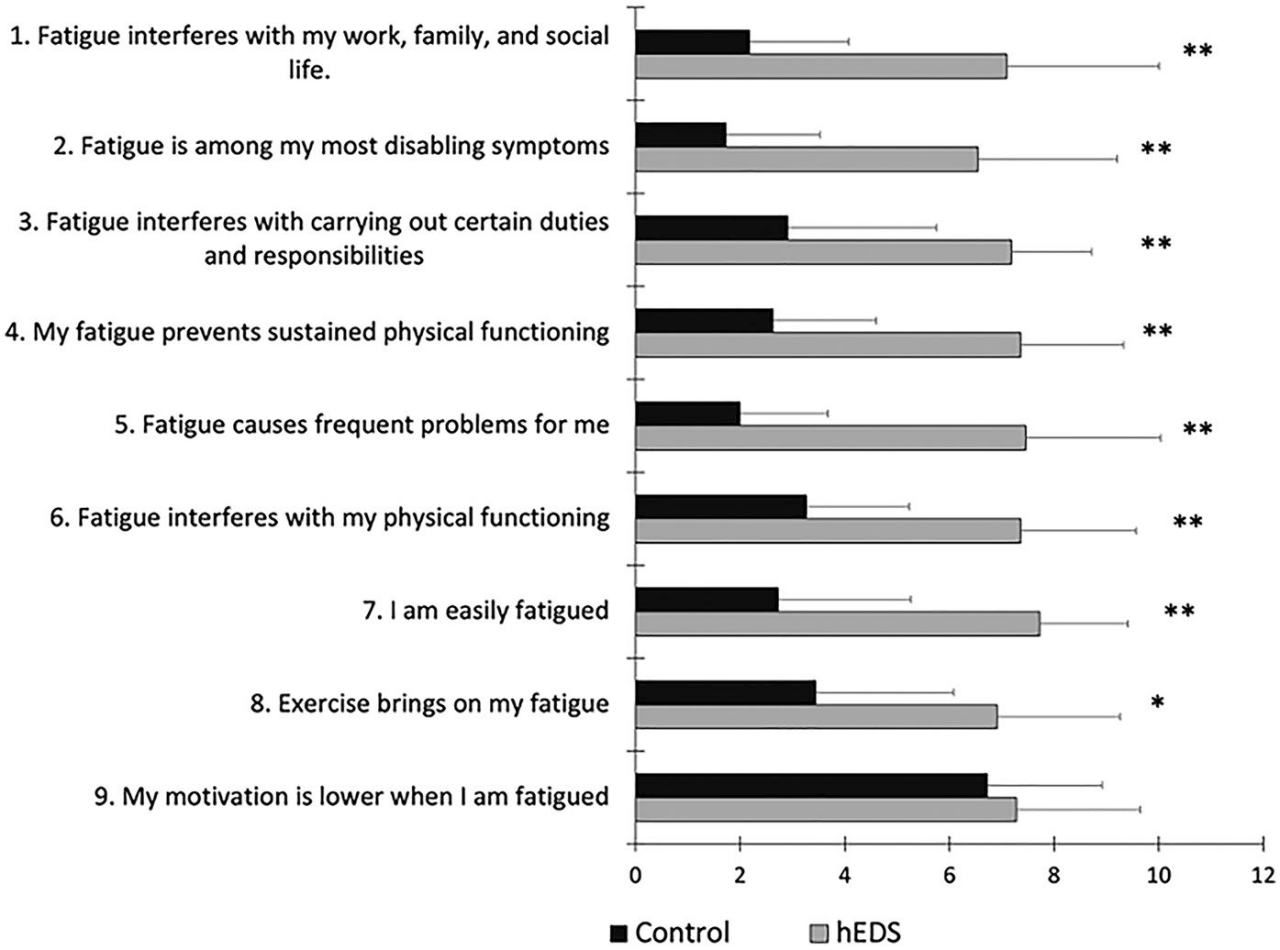
Mean ± SD FSS for HSD/hEDS (grey) and CON (black). *indicate significant group differences (*p* < 0.01), **indicate significant group differences (*p* < 0.001).

The sum FSS score was significantly higher in HSD/hEDS (64.9 ± 14.8) compared to CON (24.5 ± 10.0, *p* < 0.0001); HSD/hEDS rated their level of fatigue on the FSS significantly worse in all aspects of fatigue (*p* < 0.01, Figure 2). The lone exception was for “*my motivation is lower when I am fatigued”* which was not different between groups (*p* = 0.58). Baseline global fatigue was significantly higher in HSD/hEDS (5.0 ± 2.0) compared to CON (7.9 ± 2.9, *p* = 0.013).

**Figure 3:**
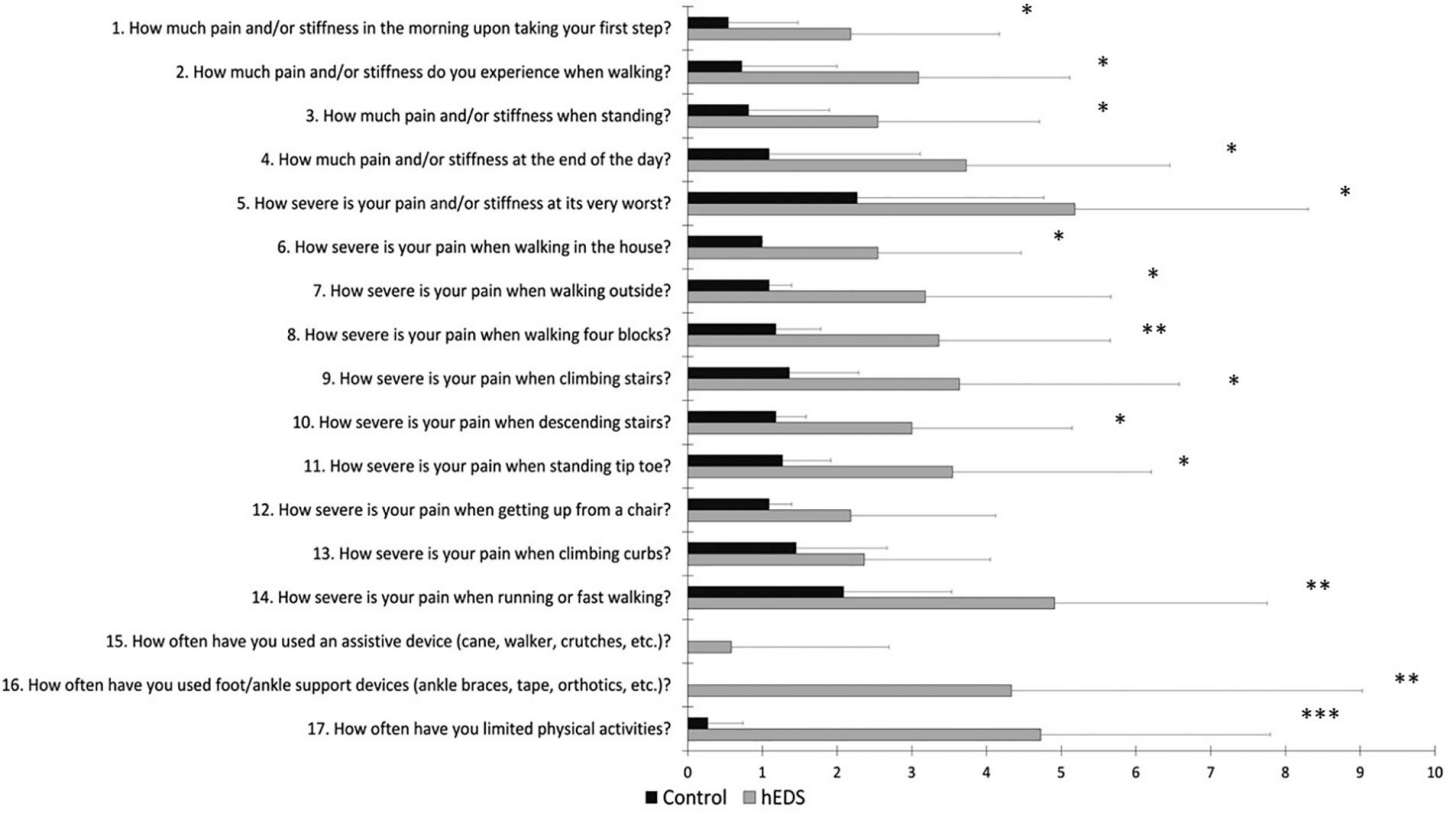
Mean ± SD FFI for HSD/hEDS (grey) and CON (black). *indicate significant group differences (*p* < 0.05), **indicate significant group differences (*p* < 0.01), *** indicate significant group differences (*p* < 0.001).

Figure 3 shows self-reported, foot-specific pain and disability, assessed by the FFI. Higher scores on the FFI indicate greater pain and disability. HSD/hEDS reported significantly higher pain (*p* < 0.05) in most aspects of the FFI.

As described previously by Sheehan et al. (20) and shown in Table 2, CoT was significantly higher in HSD/hEDS compared to CON at all speeds (*p* = 0.034). CoT was significantly higher at 80% PWS compared to both 100% and 120% PWS in HSD/hEDS (*p* < 0.034) whereas CoT was similar across speeds in CON (*p* > 0.99).

**Table 2:** Cost of transport at 80, 100 and 120% preferred walking speed (PWS).

Group	80% PWS		100% PWS		120% PWS	
	CON	HSD/hEDS	CON	HSD/hEDS	CON	HSD/hEDS
*n* =	11	11	11	11	11	11
Mean	3.74	4.77*	3.51	4.17^a^*	3.48	3.99^a^*
SD	0.70	1.27	0.64	0.67	0.69	0.80

* Significant main effect of group.

a Significantly different from 80% PWS. Data originally presented in, and modified in its present form from (20).

**Figure 4:**
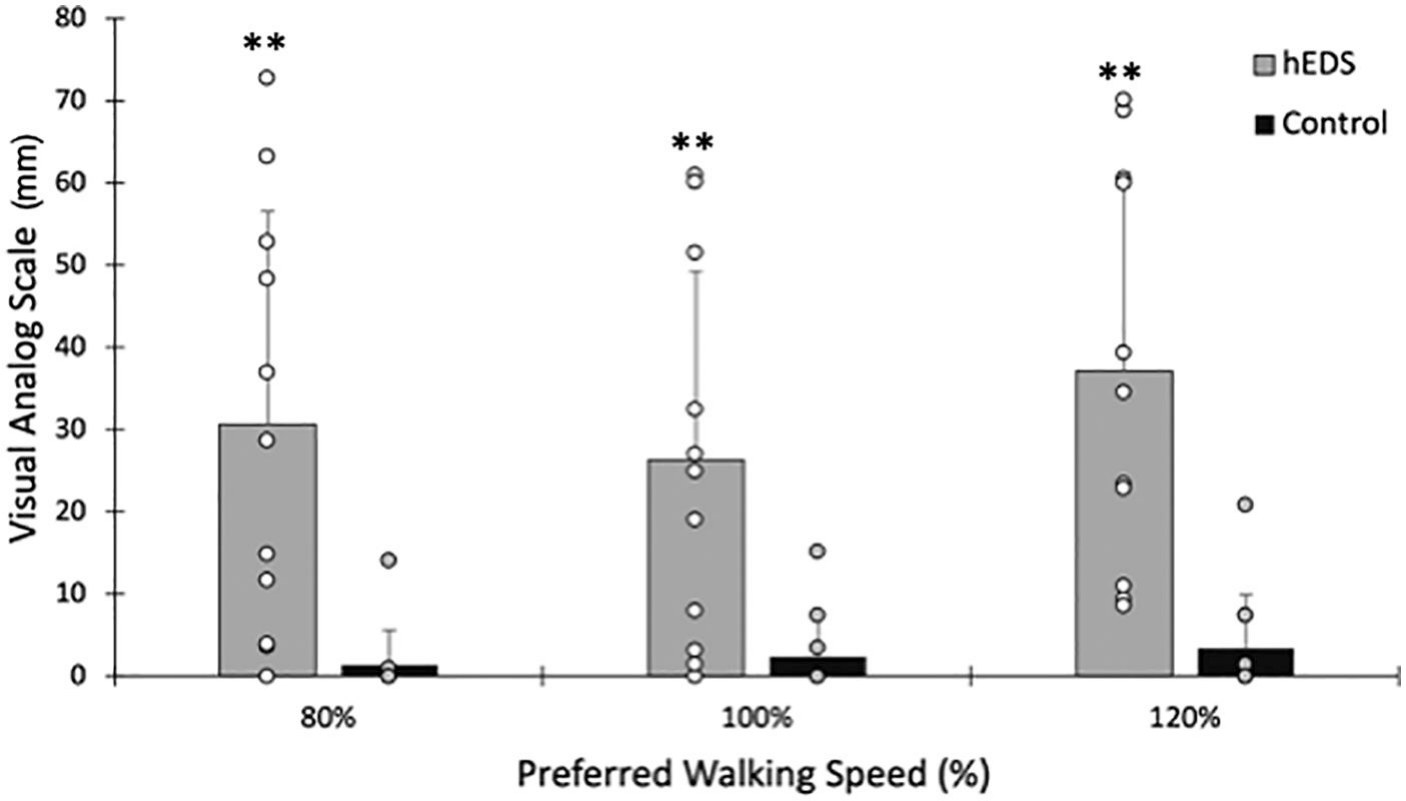
Pain following walking at 80%, 100% and 120% preferred walking speed. Mean ± SD HSD/hEDS and CON are shown as grey and black bars, respectively. Individual participant ratings are shown as open or filled circles for hEDS and CON, respectively. *indicate significant group differences (*p* < 0.05), **indicate significant group differences (*p* < 0.01), *** indicate significant group differences (*p* < 0.001).

Leg pain/fatigue immediately following walking was significantly higher in HSD/hEDS compared to CON (group main effect *p* < 0.001). A significant main effect of speed in HSD/hEDS was also demonstrated (*p* = 0.011, Figure 4); Leg pain/fatigue was significantly higher at 120% PWS compared to either 80% or 100% PWS (*p* < 0.041). In contrast, leg pain/fatigue remained constant across speeds (*p* = 0.173) and was not significantly different from “no pain” (i.e., VAS = 0 mm, *p* > 0.08 at all speeds) in CON. Relative to lower leg pain/fatigue assessed at 100% PWS, changes in lower leg pain/fatigue in HSD/hEDS was not related to the change in CoT at either 80% PWS (*r* = 0.48, *p* = 0.16) or 120% PWS (*r* = 0.1, *p* = 0.77). Because 10/11 CON participants rated no pain across all speeds, this precluded us from presenting this correlational analysis.

**Figure 5:**
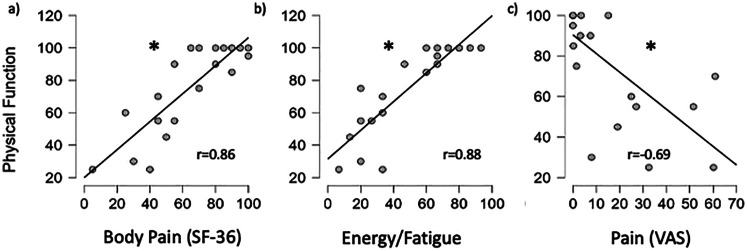
Relationship between physical function vs. body pain **(a)**, energy/fatigue **(b)** and VAS after PWS walking **(c)** for all participants. *significant relationship (*p* < 0.05).

A significant positive relationship between physical function and body pain (*r* = 0.86, *p* < 0.001) and between physical function and energy/fatigue (r = 0.88, *p* < 0.001) was seen, suggesting a higher level of physical function was related to less body pain and less energy/fatigue (Figure 5). A significant negative relationship between physical function and ratings of pain following PWS walking (*r* = −0.69, *p* < 0.001) suggests a lower physical function was associated with higher self-reported pain following walking at PWS.

## Discussion

This study aimed to determine if self-reported pain was exacerbated in individuals with HSD/hEDS following walking. A secondary purpose was to quantify the relationship between self-perceived quality of life and subjective fatigue prior to walking and lower leg pain/fatigue following walking. We found that participants with HSD/hEDS reported significantly worse levels of physical functioning, higher levels of physical role limitation, increased pain, and higher fatigue *prior to* exercise. These self-report levels of baseline pain and/or fatigue made prior to exercise may manifest themselves as higher levels of pain and/or fatigue following walking, which we show here is exacerbated with increased walking speed above the preferred speed. HSD/hEDS participants also rated their fatigue severity higher than individuals without HSD/hEDS following walking. Together, this may suggest that HSD/hEDS participants are less likely to adopt physical activity or exercise programs due to their fatigue severity and lower physical function, even prior to performing exercise. Indeed, fatigue is considered one of the largest contributing factors in an HSD/hEDS individual's self-reported deteriorating quality of life (28). Our correlational analysis at least partially supports this notion.

The HSD/hEDS participants also reported a higher metabolic cost to walk at the same walking speed relative to preferred, and a higher level of pain after walking (20). While it would be tempting to suggest that reducing CoT may reduce pain and fatigue in HSD/hEDS, since a reduced CoT can directly or indirectly be attributed to reduced muscle activations during locomotion (29, 30), this was not the case in our sample of HSD/hEDS individuals. The significant relationships found between physical function, pain, and energy/fatigue may also suggest improvements in physical function (through, for example, tailored exercise interventions) may improve upon these symptoms in individuals with HSD/hEDS.

The results of this study support the notion that individuals with HSD/hEDS have perceived their physical impairments to be severe and these impairments impact their ability to function in their normal roles (31). Castori et al. (32) also found that in a sample of HSD/hEDS patients, they differed significantly to the general population in the physical, social and emotional domains of quality of life, as assessed by the SF-36.

Celletti et al. (33) had previously reported that the magnitude of fatigue (from the FSS) was negatively related to the peak vertical ground reaction force during the push-off phase of walking at self-selected speeds, suggesting that subjective fatigue reduces self-selected walking speed. The FSS has yet to be quantified in the context of other health-related quality of life surveys such as the SF-36 and FFI and has not been analyzed alongside the quantification of pain using the VAS. This study found that there are similarly reported impairments due to fatigue as reported in both the SF-36 and the FSS, and that pain is also significantly increased in both the VAS and the SF-36 in the hEDS population.

The present study also expands upon the findings of others, showing that this self-reported higher physical impairment, and greater fatigue further present during exercise. We show that despite allowing hEDS participants to choose their preferred walking speed, this speed was not significantly slower than the preferred walking speed of individuals without hEDS. Interestingly, hEDS participants did not choose a slower PWS in order to reduce their self-reported pain of walking. Indeed, even at walking speeds 20% slower than their preferred lower leg pain/fatigue was not different than the lower leg pain/fatigue reported at higher walking speeds. This pain was also substantial (30/100) and significantly higher than the lower leg pain/fatigue in individuals without hEDS. Together, these results demonstrate the importance of considering joint hypermobility in even the most preferred intensities of exercise for these individuals.

The results of the FFI suggest that the hEDS population struggles greatly with pain in the lower leg, and that ankle stability and joint strength should be a focus of exercise-based interventions. Indeed, rehabilitation programs for individuals with HSD/hEDS have shown to be efficacious in improving exercise capacity, reduce fatigue and improve quality of life, often though without reductions in reported pain (34, 35). Exercise and rehabilitation guidelines for HSD/hEDS participants have suggested a modified (0–10) rating of perceived exertion for aerobic exercise in the moderate to hard (3–6/10) range (14). Our current lower leg pain/fatigue data suggests this may elicit an intensity where pain/fatigue is not necessarily the lowest. The current data suggests that people with hEDS experience less pain after walking at their preferred walking speed, compared to faster (120% PWS) walking speed. Together, these findings suggest that walking-based interventions should be focused at their most comfortable walking speed to minimize lower leg pain/fatigue and to promote exercise program adherence long-term.

### Limitations

This study consisted of a relatively young, and otherwise healthy sample of individuals with HSD/hEDS. These individuals were age- and sex-matched with a sample of otherwise healthy individuals without HSD/hEDS. Despite this relatively healthy sample of HSD/hEDS participants, we were still able to demonstrate significant and meaningful differences in self-reported lower leg pain/fatigue, quality of life and function between groups. We would anticipate these differences in quality of life, pain and function to be larger if we were to have recruited an older, less-functional group of individuals with hEDS.

In addition, self-reported lower leg pain/fatigue was ∼3-4× higher following walking at various walking speeds slower and faster than self-selected preferred walking speed. These reported differences in pain/fatigue between groups was evident after as little as 6 min of steady-state walking at each speed. We would suspect these self-reported pain/fatigue symptoms would be amplified in individuals with HSD/hEDS had we measured pain/fatigue following a longer walking trial (e.g., >30 min). These findings highlight the importance of improving physical function to alleviate pain symptoms in HSD/hEDS individuals as they can manifest themselves even after short bouts of physical activity. We recognize, however, that while participants were specifically instructed to self-report lower leg pain/fatigue *as a result of the walking trial*, a higher baseline fatigue, assessed together or independently from the SF-36 subscale(s) and/or the global baseline fatigue assessment, may have contributed to a higher self-reported lower leg pain/fatigue following each walking trial. We also showed significant negative relationships between physical function and self-reported lower leg pain/fatigue, both prior to and following walking (see Figure 5). We also showed a higher physical function was associated with a higher level of energy measured prior to exercise. These relationships, however, do not imply causation and additional studies are required to confirm whether elevating physical function (specifically through targeted exercise interventions aimed at building and maintaining muscle strength) can improve self-reported decrements of pain, fatigue and quality of life in HSD/hEDS individuals. Finally, due to the adaptation of the subjective scales used in this study, the reliability and validity of the modified SF-36 used here may have been altered.

## Conclusion

Our HSD/hEDS population reported significantly lower physical functioning and energy, and significantly higher aspects of pain, role limitations and foot impairments. Decreased physical function reported from the SF-36 was also associated with elevated pain and fatigue following walking. Health-related quality of life and considerations for reducing pain are important considerations for exercise prescription in HSD/hEDS. We recommend that health-related quality of life surveys are considered when completing quantitative health assessments in the HSD/hEDS population. Future directions include investigating exercise interventions for people living with HSD/hEDS that will specifically increase muscle strength and joint stability to reduce pain and fatigue during exercise and activities of daily living to improve quality of life in this population.

## Data Availability

The raw data supporting the conclusions of this article will be made available by the authors, without undue reservation.
