# Supplementary material for: Perceived quality of life, fatigue and the metabolic cost of walking in generalized hypermobility spectrum disorder and hypermobile Ehlers-Danlos syndrome
Source: Front Rehabil Sci. 2025 Nov 28;6:1706912. doi: 10.3389/fresc.2025.1706912 (PMC12698607; doi:10.3389/fresc.2025.1706912)
Supplement: Supplementary file 1 [file Datasheet1.pdf]

SF-36 Adapted Health Survey

General Health

1. In general, would you say your health is:

- ◯ Excellent
- ◯ Very good
- ◯ Good
- ◯ Fair
- ◯ Poor

2. Compared to one year ago, how would you rate your health in general now?

- ◯ Much better now than one year ago
- ◯ Somewhat better now that one year ago
- ◯ About the same
- ◯ Somewhat worse now that one year ago
- ◯ Much worse now than one year ago

3. I seem to get sick a little easier than other people

- ◯ Definitely true
- ◯ Mostly true
- ◯ Don't know
- ◯ Mostly false
- ◯ Definitely false

4. I am as healthy as anybody I know

- ◯ Definitely true
- ◯ Mostly true
- ◯ Don't know
- ◯ Mostly false
- ◯ Definitely false

5. I expect my health to get worse

- ◯ Definitely true
- ◯ Mostly true
- ◯ Don't know
- ◯ Mostly false
- ◯ Definitely false

6. My health is excellent

- ◯ Definitely true
- ◯ Mostly true
- ◯ Don't know
- ◯ Mostly false
- ◯ Definitely false

Limitations of Activities

The following items are about activities you might do during a typical day. Does your health now limit you in these activities? If so, how much? 0 (no, not limited at all), 1 (yes, limited a little), 2 (yes, limited a lot)

7. Vigorous activities, such as running, lifting heavy objects, participating in strenuous sports

- ◯ 0 - No, not limited at all
- ◯ 1
- ◯ 2 - Yes, limited a lot

8. Moderate activities, such as moving a table, pushing a vacuum cleaner, bowling,or playing golf

- ◯ 0 - No, not limited at all
- ◯ 1
- ◯ 2 - Yes, limited a lot

9. Lifting or carrying groceries

- ◯ 0 - No, not limited at all
- ◯ 1
- ◯ 2 - Yes, limited a lot

10. Climbing several flights of stairs

- ◯ 0 - No, not limited at all
- ◯ 1
- ◯ 2 - Yes, limited a lot

11. Climbing one flight of stairs

- ◯ 0 - No, not limited at all
- ◯ 1
- ◯ 2 - Yes, limited a lot

12. Bending, kneeling, stooping

- 0 - No, not limited at all
- 1
- 2 - Yes, limited a lot

13. Walking more than a mile

- 0 - No, not limited at all
- 1
- 2 - Yes, limited a lot

14. Walking several blocks

- 0 - No, not limited at all
- 1
- 2 - Yes, limited a lot

15. Walking one block

- 0 - No, not limited at all
- 1
- 2 - Yes, limited a lot

16. Bathing or dressing yourself

- 0 - No, not limited at all
- 1
- 2 - Yes, limited a lot

17. Keeping balance

- ◯ 0 - No, not limited at all
- ◯ 1
- ◯ 2 - Yes, limited a lot

Physical Health Problems

During the past 4 weeks, have you had any of the following problems with your work or other regular daily activities as a result of your physical health?

18. Cut down the amount of time you spent on work or other activities

- ◯ Yes
- ◯ No

19. Accomplished less than you would have liked

- ◯ Yes
- ◯ No

20. Were limited in the kind of work or other activities

- ◯ Yes
- ◯ No

21. Had difficulty performing the work or other activities (for example, it took extra effort)

- ◯ Yes
- ◯ No

Pain

22. How much bodily pain have you had during the past 4 weeks?

0	1	2	3	4	5	6	7	8	9	10

None　　Very Severe

23. During the past 4 weeks, how much did pain interfere with your normal work (including both work outside the home and housework)?

0	1	2	3	4	5	6	7	8	9	10

None　　Very Severe

Energy and Emotions

24. I have a lot of energy

- ◯ All of the time
- ◯ Most of the time
- ◯ A good bit of the time
- ◯ Some of the time
- ◯ A little bit of the time
- ◯ None of the time

25. I am fearful that I will injure myself

- ◯ All of the time
- ◯ Most of the time
- ◯ A good bit of the time
- ◯ Some of the time
- ◯ A little bit of the time
- ◯ None of the time

26. I feel worn out

- ◯ All of the time
- ◯ Most of the time
- ◯ A good bit of the time
- ◯ Some of the time
- ◯ A little bit of the time
- ◯ None of the time

27. I am a happy person

- All of the time
- Most of the time
- A good bit of the time
- Some of the time
- A little bit of the time
- None of the time

28. I feel discouraged about my physical health

- All of the time
- Most of the time
- A good bit of the time
- Some of the time
- A little bit of the time
- None of the time

Social Activities

29. During the past 4 weeks, how much of the time has your physical health oremotional problems interfered with your social activities (socializing with friends,relatives, etc,)?

- ◯ All of the time
- ◯ Most of the time
- ◯ Some of the time
- ◯ A little bit of the time
- ◯ None of the time
