# Supplementary material for: Perceived quality of life, fatigue and the metabolic cost of walking in generalized hypermobility spectrum disorder and hypermobile Ehlers-Danlos syndrome
Source: Front Rehabil Sci. 2025 Nov 28;6:1706912. doi: 10.3389/fresc.2025.1706912 (PMC12698607; doi:10.3389/fresc.2025.1706912)
Supplement: Supplementary file 2 [file Datasheet2.pdf]

Fatigue Severity Scale

Please select the number that best indicates your experience within the last week (7 days)

* Required

1. My motivation is lower when I am fatigued *

Mark only one oval.

	1	2	3	4	5	6	7	8	9	10	
Strongly disagree											Strongly agree

2. Exercise brings on my fatigue *

Mark only one oval.

	1	2	3	4	5	6	7	8	9	10	
Strongly disagree											Strongly agree

3. I am easily fatigued *

Mark only one oval.

	1	2	3	4	5	6	7	8	9	10	
Strongly disagree											Strongly agree

4. Fatigue interferes with my physical functioning *

Mark only one oval.

	1	2	3	4	5	6	7	8	9	10	
Strongly disagree											Strongly agree

5. Fatigue causes frequent problems for me *

Mark only one oval.

	1	2	3	4	5	6	7	8	9	10	
Strongly disagree											Strongly agree

6. My fatigue prevents sustained physical functioning *

Mark only one oval.

	1	2	3	4	5	6	7	8	9	10	
Strongly disagree											Strongly agree

7. Fatigue interferes with carrying out certain duties and responsibilities *

Mark only one oval.

	1	2	3	4	5	6	7	8	9	10	
Strongly disagree											Strongly agree

8. Fatigue is among my most disabling symptoms *

Mark only one oval.

	1	2	3	4	5	6	7	8	9	10	
Strongly disagree											Strongly agree

9. Fatigue interferes with my work, family, and social life. *

Mark only one oval.

	1	2	3	4	5	6	7	8	9	10	
Strongly disagree	⬭	⬭	⬭	⬭	⬭	⬭	⬭	⬭	⬭	⬭	Strongly agree

Visual Analogue Fatigue Scale (VAFS)

10. Please select on the number line, which describes your global fatigue with 0 being worst and 10 being normal *

Mark only one oval.

	0	1	2	3	4	5	6	7	8	9	10	
Worst fatigue	⬭	⬭	⬭	⬭	⬭	⬭	⬭	⬭	⬭	⬭	⬭	Normal

This content is neither created nor endorsed by Google.

Google Forms
